# Markers of Mitochondrial Injury and Neurological Outcomes of Comatose Patients after Cardiac Arrest

**DOI:** 10.3390/medicina60081286

**Published:** 2024-08-09

**Authors:** Ina Živanović, Katarina Miš, Sergej Pirkmajer, Ivica Marić, Tomaž Goslar

**Affiliations:** 1 Department of Intensive Internal Medicine, University Medical Centre Ljubljana, Zaloska cesta 7, 1000 Ljubljana, Slovenia; tomaz.goslar@kclj.si; 2 Faculty of Medicine, University of Ljubljana, Vrazov trg 2, 1000 Ljubljana, Slovenia; 3 Institute of Pathophysiology, Faculty of Medicine, University of Ljubljana, Zaloska cesta 4, 1000 Ljubljana, Slovenia; 4 Blood Transfusion Centre of Slovenia, Slajmerjeva 6, 1000 Ljubljana, Slovenia

**Keywords:** cardiac arrest, neuroprognostication, mitochondria, cytochrome c, mtDNA

## Abstract

*Background and Objectives*: Most patients who are successfully resuscitated from cardiac arrest remain comatose, and only half regain consciousness 72 h after the arrest. Neuroprognostication methods can be complex and even inconclusive. As mitochondrial components have been identified as markers of post-cardiac-arrest injury and associated with survival, we aimed to investigate cytochrome c and mtDNA in comatose patients after cardiac arrest to compare neurological outcomes and to evaluate the markers’ neuroprognostic value. *Materials and Methods*: This prospective observational study included 86 comatose post-cardiac-arrest patients and 10 healthy controls. Cytochrome c and mtDNA were determined at admission. Neuron-specific enolase (NSE) was measured after 72 h. Additional neuroprognostication methods were performed when patients remained unconscious. Cerebral performance category (CPC) was determined. *Results*: Cytochrome c was elevated in patients compared to healthy controls (2.029 [0.85–4.97] ng/mL vs. 0 [0.0–0.16], *p* < 0.001) but not mtDNA (95,228 [52,566–194,060] vs. 41,466 [28,199–104,708] copies/μL, *p* = 0.074). Compared to patients with CPC 1–2, patients with CPC 3–5 had higher cytochrome c (1.735 [0.717–3.40] vs. 4.109 [1.149–8.457] ng/mL, *p* = 0.011), with no differences in mtDNA (87,855 [47,598–172,464] vs. 126,452 [69,447–260,334] copies/μL, *p* = 0.208). Patients with CPC 1–2 and CPC 3–5 differed in all neuroprognostication methods. In patients with good vs. poor neurological outcome, ROC AUC was 0.664 (*p* = 0.011) for cytochrome c, 0.582 (*p* = 0.208) for mtDNA, and 0.860 (*p* < 0.001) for NSE. The correlation between NSE and cytochrome c was moderate, with a coefficient of 0.576 (*p* < 0.001). *Conclusions*: Cytochrome c was higher in comatose patients after cardiac arrest compared to healthy controls and higher in post-cardiac-arrest patients with poor neurological outcomes. Although cytochrome c correlated with NSE, its neuroprognostic value was poor. We found no differences in mtDNA.

## 1. Introduction

Out-of-hospital cardiac arrest (OHCA) is one of the most common causes of death in Europe, with an incidence of 56 per 100,000 annually [1,2]. Furthermore, in-hospital cardiac arrest (IHCA) occurs in 1.5–2.8 out of 1000 hospital admissions [3]. A total of 81% of patients after OHCA remain comatose [4], and only half wake up 72 h after the arrest [5]. The extent of hypoxic–ischemic brain injury is assessed using multimodal neuroprognostic methods that support decision-making regarding life-sustaining measures. However, neuroprognostication can be complex and suboptimally sensitive, often leading to an indeterminate prognosis, long observation times, and even futile treatment [6,7,8].

Mitochondria are at the center of ischemic–reperfusion injury in cardiac arrest and post-cardiac-arrest syndrome [9,10]. They show organ-specific vulnerability and responses, have both protective and damaging properties, and represent potential therapeutic targets. Mitochondrial components have a multitude of functions. Cytochrome c is an inner-membrane mitochondrial hemoprotein involved in cellular respiration, antioxidation, apoptosis, and inflammation [11]. Mitochondrial DNA (mtDNA) is a circular DNA encoding polypeptides and RNAs in the mitochondria [12]. Both cytochrome c and mtDNA have roles in pro- and anti-inflammatory processes and cell death. Because mitochondria are derived from bacterial endosymbionts, mtDNA resembles bacterial DNA and acts as an inflammatory trigger, a damage-associated molecular pattern (DAMP) [13,14]. Cytochrome c induces apoptosis when it is dislocated into the cytoplasm and inflammation when it is dislocated into the extracellular space [13]. The greater the tissue damage, the higher the levels of mitochondrial components.

In animals, mitochondrial components released into the circulation have been identified as markers of post-cardiac-arrest injury and predictors of survival [15]. Studies in patients after OHCA also showed elevated mitochondrial markers with significant differences between survivors and non-survivors. Cytochrome c was high in patients after OHCA and higher in subsequent non-survivors [16]. MtDNA was also increased in non-survivors after OHCA, with excellent discrimination for 3-day survival [17]. Extrapolating from studies so far, higher levels of cytochrome c and mtDNA may be associated with longer cardiac arrest duration and greater tissue damage due to ischemia–reperfusion or other causes. The brain is a highly aerobic organ with high energy demands, rich in mitochondria and particularly susceptible to ischemia–reperfusion injury [10]. Increased mtDNA has been demonstrated in acute ischaemic stroke as well as bacterial meningitis [18,19]. However, knowledge on the possible utilization of mitochondrial markers in hypoxic–ischemia brain injury after cardiac arrest is scarce. The aim of our study was to investigate whether cytochrome c and mtDNA are associated with neurological outcomes in comatose patients after cardiac arrest and whether they can be used to facilitate and improve neuroprognostication.

## 2. Materials and Methods

### 2.1. Study Design and Participants

The prospective observational study included 96 patients who remained comatose after cardiac arrest and were admitted to the Department of Intensive Internal Medicine, University Medical Centre Ljubljana, Slovenia, between 2019 and 2023. The inclusion criteria for patients were as follows: (1) comatose state after cardiac arrest, defined as the patient being unconscious and unresponsive, with a Glasgow Coma Scale (GCS) ≤ 8; (2) age over 14 years; (3) treatment with active temperature control. An exclusion criterion was a moribund state—more precisely, if the patient was expected to die within 24 h. The local intensive care unit (ICU) protocol for comatose after-cardiac arrest patients consisted of the following: (a) active temperature control (33–36 ± 0.5 °C for 48 h, including rewarming); (b) analgosedation (Richmond agitation sedation scale (RASS)-4, patient state index (PSI) below 20–30, or bispectral index (BIS) 20–40), which was weaned after 48 h unless the patient’s condition required prolongation; and (c) additional neuroprotective measures (head elevation to 30°, mechanical ventilation ensuring normoxemia and normocapnia, glucose and electrolyte control, and prevention of central temperature above 37.4 °C after completion of normothermia up to 72 h after admission). Standard neuroprognostication was performed at 72–96 h after return of spontaneous circulation (ROSC). In addition to the active temperature control protocol, post-resuscitation care and treatments were provided according to the individual needs of the patient per the attending physicians’ discretion.

**Figure 1:**
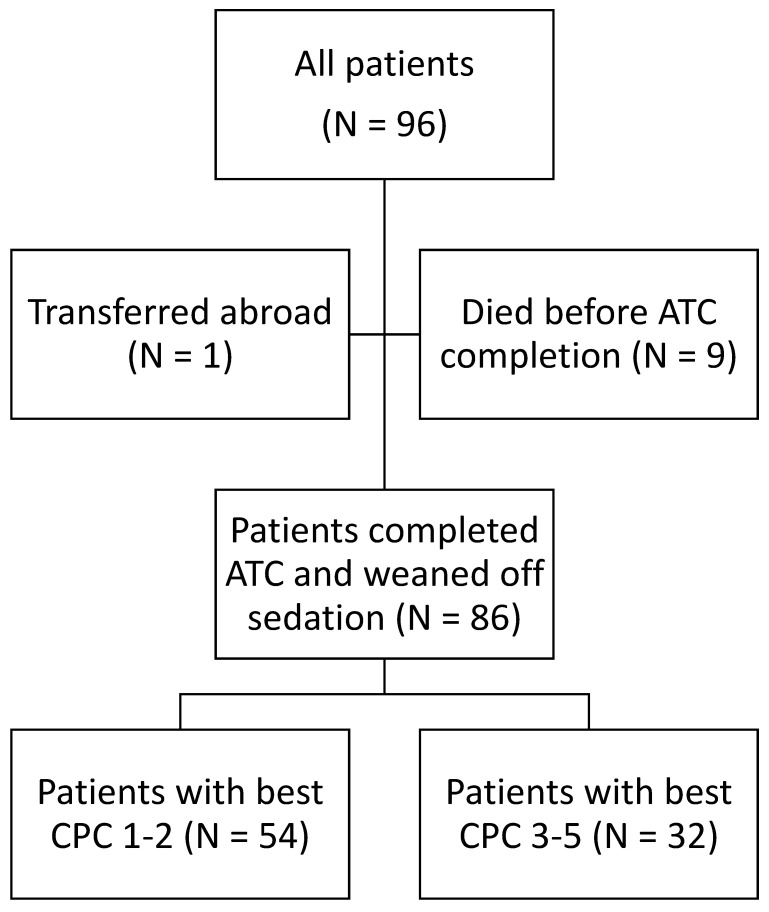
Flowchart. ACT = active temperature control; CPC = cerebral performance category.

Of the 96 patients included, 9 patients died before completing the active temperature control protocol and being weaned off sedation, and 1 patient was transferred abroad before being weaned off sedation. Therefore, the final sample consisted of 86 patients, which is presented in the flowchart (Figure 1). Ten control subjects without cardiac arrest aged over 14 years were also included.

This study was approved by the National Medical Ethics Committee of the Republic of Slovenia (approval number 0120-78/2018/3). A waiver of informed consent was obtained as patients were unable to consent due to impaired consciousness, and inclusion in the study would not affect treatment or health status. Informed consent was obtained from the participants in the control group. This study was registered at ClinicalTrials.gov (ID NCT03539497, accessed on 14 July 2024).

### 2.2. Neurological Outcome Assessment

Patients’ neurological outcomes after cardiac arrest are most commonly assessed using the cerebral performance category (CPC) score [6], which is also part of the local ICU protocol. It is categorized as follows: no or minimal neurological disability (CPC 1); minor neurological disability (CPC 2); severe neurological disability (CPC 3); persistent vegetative state (CPC 4); brain death and death (CPC 5). A good neurological outcome is defined as CPC 1–2 and a poor neurological outcome as CPC 3–5. CPC score was determined both in the ICU and prior to hospital discharge. The patients’ best CPC scores were used for the analysis to avoid the effect of a possible decision to not escalate life-sustaining treatment per the attending physicians’ discretion for patients with severe hypoxic–ischemic brain injury, or death due to another cause during hospitalization.

### 2.3. Neuroprognostication Methods

In addition to clinical assessment with determination of a CPC score, the standard methods of neuroprognostication for post-cardiac-arrest patients consisted of neuron-specific enolase (NSE) concentration, computed tomography (CT) of the head, electroencephalography (EEG), and somatosensory evoked potentials (SSEP) [6,8]. The NSE concentration was determined 72 h after ROSC. CT of the head, EEG, and SSEP were performed 72–96 h after ROSC if the patient did not regain consciousness after weaning from analgosedation. Head CT results were categorized as (a) normal, (b) with signs of mild hypoxic–ischemic brain injury, or (c) with signs of moderate to severe hypoxic–ischemic brain injury. EEG results were categorized as either not very malignant or very malignant. The latter included (a) suppressed basic activity with an amplitude < 10 µV, (b) suppressed basic activity < 10 µV with continuous periodic graphoelements appearing in >90% of the recording, and (c) markedly discontinuous activity with suppressed basic activity <10 µV in >50% of the recording and possible spikes and sharp waves (burst–silence). SSEP results were categorized as an absence or presence of cortical SSEP (N20).

### 2.4. Analysis of Mitochondrial Markers

The blood samples for the determination of cytochrome c and mtDNA serum concentrations were collected at admission to the ICU. The samples were then transported to the Blood Transfusion Center of Slovenia, centrifuged, aliquoted into four vials, and stored at −80 °C. Serum cytochrome c was measured via enzyme-linked immunosorbent assay (ELISA) and serum mtDNA via quantitative polymerase chain reaction (qPCR) at the Institute of Pathophysiology, Faculty of Medicine, University of Ljubljana.

### 2.5. ELISA

Cytochrome c measurements were performed using the Cytochrome C Human ELISA Kit from Thermo Fisher Scientific (Waltham, MA, USA) in strict accordance with the manufacturer’s protocol, including serum dilution recommendation, i.e., 2-fold in assay buffer. Absorbance at 450 nm and 620 nm as reference were measured on a BioTek Epoch microplate spectrophotometer (Agilent Technologies, Santa Clara, CA, USA). The concentration range in the standard curve was 0.08–5 ng/mL. A 5-parameter curve fit was used for analyses.

### 2.6. Copy Number of Circulating Cell-Free Mitochondrial DNA (ccf mtDNA)

Due to the low quantity of mtDNA in serum samples, we used low DNA-binding plastic consumables (vials, Eppendorf and pipet filter tips, Gilson) and DNase/RNase-free water supplemented with 25 µg/mL polyA-DNA (Carrier DNA; MP Biomedicals) to avoid possible loss of mtDNA. Total DNA was isolated and purified using QIAamp DNA Blood Mini Kit (Qiagen, Venlo, The Netherlands) [20] according to the manufacturer´s protocol with the following modifications: the serum samples were thawed just before use, mixed, and centrifuged at 4 °C and 18,000× *g* for 15 min. A total of 200 µL of serum supernatants cleared of cells was further processed with 20 µL of protease and 200 µL of lysis buffer AL (both from the kit), previously supplemented with carrier DNA (25 µg/mL). After all the following column separation and washing steps, DNA was eluted with centrifugation after 5 min in-column incubation with 60 µL of DNase/RNase-free water. For the assessment of total mtDNA quantity, mitochondrially encoded NADH dehydrogenase 1 (MT-ND1) was selected as one of the most stable mitochondrial genes [21]. Absolute quantification of MT-ND1 was performed on a QuantStudio™ 3 qPCR System (Thermo Fisher Scientific) using the TaqMan chemistry. TaqMan Universal Master Mix, TaqMan gene expression assay for human MT-ND1 (Assay ID: Hs02596873_s1), MicroAmp optical 96-well reaction plates, and MicroAmp optical adhesive sheets were obtained from Thermo Fisher Scientific (Waltham, MA, USA). As a standard, a custom-made gBlock (Integrated DNA technologies, IDT) [22] gene fragment was synthesized with the same size and the same sequence as qPCR amplicon of native MT-ND1. A total of 79.8 fmol/µL with 6 additional 10-fold dilutions was used for the standard curve. ccf mtDNA copy number/µL was calculated via Quant Studio Design and Analysis Software 1.5.2.

### 2.7. Statistical Analysis

Continuous data are presented according to their distribution as mean with standard deviation or median with interquartile range, and categorical data are presented as number of subjects and percentage. Patient characteristics were compared using Student’s *t*-test for independent samples or Mann–Whitney U-test for continuous values based on their distribution and chi-square test or Fisher’s exact test for categorical values. Receiver operating characteristic (ROC) curves were generated for cytochrome c, mtDNA, and neuroprognostication studies. A Youden index was calculated for mitochondrial markers with significant differences between groups. The correlation between significant mitochondrial markers and NSE was tested using Spearman’s correlation. The level of statistical significance was set at *p* < 0.05. IBM SPSS Statistics 26 (IBM, Armonk, NY, USA) was used for the analysis.

## 3. Results

### 3.1. Patients after Cardiac Arrest Compared to Controls

Patients after cardiac arrest did not differ from the control group in terms of demographic characteristics or the Charlson Comorbidity Index (CCI) (Table 1). The cytochrome c concentration was 0 ng/mL in controls and was significantly higher at 2.029 ng/mL after cardiac arrest. The concentration of mtDNA was not significantly higher.

### 3.2. Characteristics of Patients with CPC Score 1–2 and CPC Score 3–5

Based on their neurological outcome, post-cardiac-arrest patients did not differ in their baseline demographic characteristics and CCI (Table 2). Patients with CPC score 1–2 had a higher rate of shockable rhythms and a shorter duration of cardiac arrest. There were no other differences in the type and aetiology of cardiac arrest or accessibility of emergency interventions. Patients did not differ in percutaneous coronary intervention (PCI) frequency prior to admission or troponin I (high sensitivity) levels at admission. At admission to the ICU, patients with CPC score 1–2 had lower lactate levels, Simplified Acute Physiology Score II (SAPS II) and Acute Physiology And Chronic Health Evaluation II (APACHE II) Scores. There were no differences in vasopressor or inotrope use. The temperature of the patients at admission and the active temperature control measures did not differ.

### 3.3. Mitochondrial Markers and Neuroprognostication in Patients with CPC 1–2 and CPC 3–5

**Figure 2:**
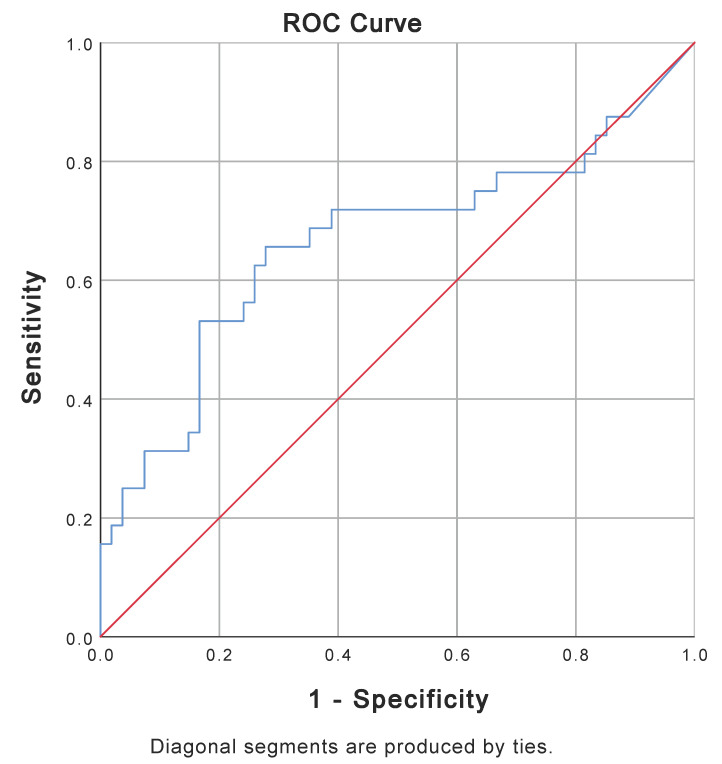
ROC curve for cytochrome c at ICU admission for patients with CPC 1–2 (N = 54) and patients with CPC 3–5 (N = 32). AUC is 0.664 (*p*-value = 0.011). AUC = area under the curve; CPC = Cerebral Performance Category Score; ICU = intensive care unit; ROC = receiver operating characteristic.

Sampling for mitochondrial markers was performed at admission to the ICU, with a median time of 2 h and 40 min [127–201 min] for all post-cardiac-arrest patients. There was no difference in sampling time between the groups of patients with CPC 1–2 and CPC 3–5 (Table 3). Patients with CPC score 1–2 had significantly lower cytochrome c levels after cardiac arrest than patients with CPC score 3–5. For the two groups, ROC analysis of cytochrome c showed an area under the curve (AUC) of 0.664 (*p* = 0.011) (Figure 2). Based on the Youden index (0.378), the cut-off value for a poor neurological outcome using cytochrome c was 2.748 ng/mL.

Despite a trend towards lower mtDNA levels in patients with a CPC score of 1–2, the difference from patients with a CPC score 3–5 was not significant (Table 3). The ROC analysis of mtDNA based on CPC groups resulted in an AUC of 0.582 (*p* = 0.208).

**Figure 3:**
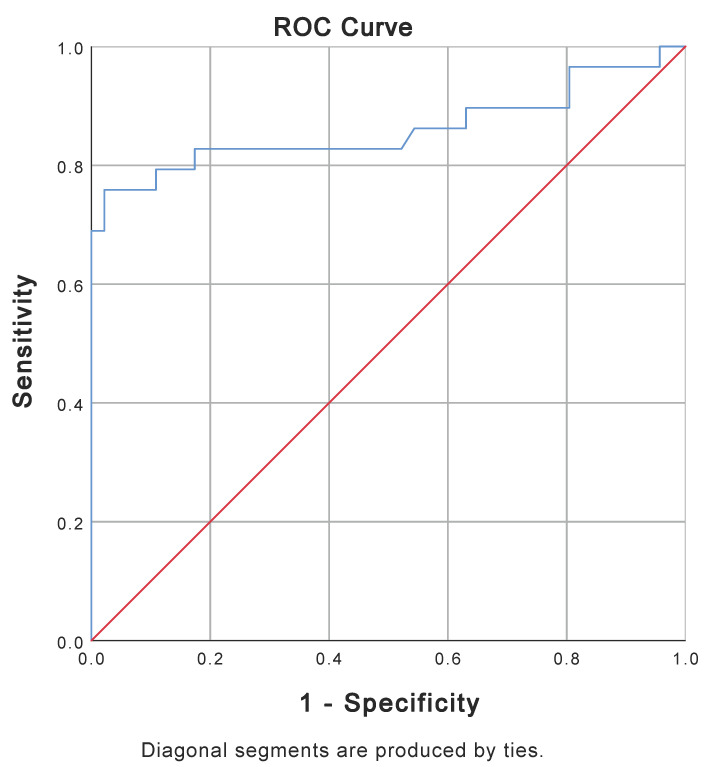
ROC curve for NSE 72 h after ROSC for patients with CPC 1–2 (N = 46) and patients with CPC 3–5 (N = 29). AUC for NSE is 0.860 (*p*-value < 0.001). Five patients with CPC 3–5 had NSE above upper the measurable limit of 370 mcg/L. AUC = area under the curve; CPC = Cerebral Performance Category Score; NSE = neuron-specific enolase; ROC = receiver operating characteristic; ROSC = return of spontaneous circulation.

**Figure 4:**
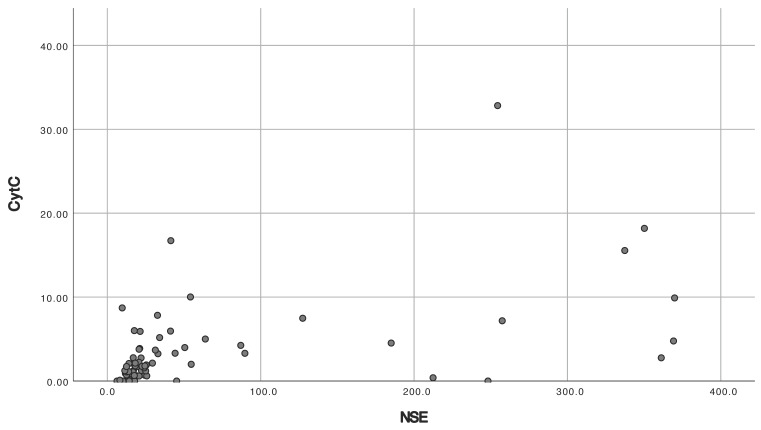
Scatterplot for cytochrome c by NSE. Five patients with CPC 3–5 had NSE above upper the measurable limit of 370 mcg/L and were excluded. CPC = Cerebral Performance Category Score; NSE = neuron-specific enolase.

Patients with a CPC score of 1–2 and a CPC score of 3–5 differed in all standard neuroprognostication studies (Table 3). Patients with good neurological outcomes had lower levels of NSE 72 h after ROSC, lower rates of hypoxic–ischemic brain injury on head CT, lower rates of very malignant EEG, and lower rates of absent cortical SSEP 72–96 h after ROSC. ROC analysis of NSE in patients with CPC scores of 1–2 and 3–5 showed an AUC of 0.860 (*p* < 0.001) (Figure 3). Spearman’s analysis showed a correlation between cytochrome and NSE (r = 0.576, *p* < 0.001) (Figure 4) and cytochrome c and lactate (r = 0.471, *p* < 0.001) and no correlation between cytochrome c and APACHE II (r = 0.145, *p* = 0.184) or SAPS II (r = 0.083, *p* = 448).

## 4. Discussion

We found that patients after cardiac arrest had increased cytochrome c compared to controls, while mtDNA was not significantly increased. Patients with poor neurological outcome (CPC score 3–5) had higher cytochrome c levels after cardiac arrest, which correlated moderately with NSE at 72 h. ROC analysis for cytochrome c showed poor discrimination between good and poor neurological outcomes with a cut-off value of 2.748 ng/mL. MtDNA was not significantly increased after cardiac arrest in patients with poor neurological outcome.

The comparison of cytochrome c between healthy individuals and patients after cardiac arrest yielded similar results to other studies in this field. A previous study in comatose adults after OHCA also reported minute cytochrome c levels in healthy controls, with a similar increase to 2.18 ng/mL after OHCA, followed by a decrease over 36 h [16]. These results underscore the importance of cytochrome c as a mitochondrial marker of ischemic–reperfusion injury in cardiac arrest. Interestingly, the first animal study of cytochrome c in cardiac arrest reported a subsequent decline to baseline levels or a progressive increase until death [15], which may reflect the absence of post-resuscitation care.

One of the first studies in this field found differences in plasma mtDNA among patients after cardiac arrest, with levels twice as high in patients who died within 24 h [17]. In contrast, a later multicenter study found no difference in mtDNA-rRNA between post-cardiac-arrest patients and controls and found even significantly lower mtDNA-D loop levels in post-cardiac-arrest patients [16]. Although we observed more than twofold higher mtDNA levels in post-cardiac-arrest patients compared to healthy controls, there was no significant difference between the two groups. The reasons for this could be small sample size, especially in the control group; differences in the release of mtDNA compared to cytochrome c due to its localization in the mitochondrial matrix, as well as specific tissue responses to ischemic–reperfusion injury; and our use of serum samples into which mtDNA from platelets is also released during coagulation [23].

When comparing patients with a CPC score of 1–2 to those with a CPC score of 3–5, we found the expected differences [24,25] in terms of the longer duration of cardiac arrest, greater frequency of non-shockable rhythms, and higher lactate, SAPS II, and APACHE II scores in the group with poor neurological outcomes. Patients with a CPC score of 3–5 had higher NSE levels 72 h after ROSC and were significantly more likely to have signs of severe hypoxic–ischemic brain injury on head CT, EEG, and SSEP 72–96 h after ROSC. ROC analysis showed that NSE was an excellent predictor of neurological outcome but with the possible bias of being a self-fulfilling prophecy [8].

Previous studies on cytochrome c after OHCA have produced inconclusive results. One smaller study found no difference between survivors and non-survivors in terms of cytochrome c after OHCA and no improvement in the model for predicting mortality [26]. The reason might have been a small sample for cytochrome c analysis, particularly in relation to the overall group size. Another study found differences between survivors and non-survivors but no association with mortality in the logistic regression and no differences between patients with neurologic and other causes of death [16]. However, the same study found higher cytochrome c levels in patients with poor functional outcome. We observed significantly higher levels of cytochrome c in patients with worse neurological outcomes after cardiac arrest. In addition, cytochrome c correlated with NSE. In contrast, ROC analysis showed poor discrimination of cytochrome c for good or poor neurological outcome, with a cut-off value of 2.748 ng/mL.

Cytochrome c could be advantageous in that it has the potential to facilitate early neuroprognostication at patient admission, potentially avoiding futile treatment. However, it is crucial that markers of neuroprognostication have high sensitivity and specificity as they are used for decisions on life-sustaining treatments, and this is a major limitation for cytochrome c according to our study. We cannot completely rule out other sources of cytochrome c as ischemic–reperfusion injury in cardiac arrest affects multiple organs [10], although the brain is particularly susceptible. Also, the underlying cause of cardiac arrest itself can be the cause of increased cytochrome c. However, both will also occur in real-world scenarios. Our study showed no significant differences when comparing groups of patients with CPC scores of 1–2 and 3–5 in terms of chronic comorbidities, causes of cardiac arrest, basic life support, and emergency medical services, nor in post-resuscitation vasoactive therapy. Since the predominant cause of cardiac arrest in our group was acute myocardial infarction, we also compared and found no differences in percutaneous coronary intervention frequency before admission and troponin at admission between patients with CPC scores of 1–2 and 3–5. SAPS II and APACHE II, which displayed intergroup differences, did not correlate with cytochrome c, while lactate did correlate with cytochrome c, albeit with a moderate coefficient. In comparison to NSE, which is a highly specific marker for neurons and neuroendocrine cells [27], cytochrome c is not. This was probably the reason why we found NSE to be an excellent predictor of neurological outcome and cytochrome c to be a poor predictor.

Studies on mtDNA in patients after OHCA have produced conflicting results as well. One study found no differences in mtDNA markers between survivors and non-survivors during a 36 h period after OHCA [16]. In another study, mtDNA was found to be an excellent predictor of 3-day mortality after OHCA, with significant differences between survivors and non-survivors [17]. We found a trend towards increased mtDNA in post-cardiac-arrest patients compared to healthy controls and in patients with poor vs. good neurological outcome. However, the difference was not significant in either case, suggesting that mtDNA might not have prognostic value for neurological outcome. As mentioned earlier, this might be due to differences in the release of mtDNA compared to cytochrome c and to the use of serum instead of plasma samples.

### 4.1. Clinical Implications

Cytochrome c shows the potential to enhance current neuroprognostic methods in comatose patients after cardiac arrest, particularly as an early marker obtainable immediately at admission. This is in contrast to current neuroprognostication methods, which are performed 48–72 h post-ROSC [8]. As an early marker, cytochrome c would be specifically useful for differentiating severe hypoxic–ischemic brain injury, which would aid clinical decision-making. Larger studies including standard neuroprognostication methods are warranted.

### 4.2. Study Limitations

While our study has the advantage of being a prospective study, it is a single-center study with a small sample size [28]. A larger study population would have increased statistical power, which would have been particularly valuable for mtDNA comparison. Furthermore, our study was an observational study conducted in a real-world setting. While this lent our study the advantage of applicability, the heterogeneous patient population (and even the discontinuation of treatment in some patients) reduced the degree of variable control and increased the degree of complexity.

## 5. Conclusions

We found increased cytochrome c levels in comatose patients after cardiac arrest compared to healthy controls and higher cytochrome c levels in patients with a subsequently poor neurological outcome. Cytochrome c also correlated with NSE, but its neuroprognostic value was poor. We found no significant differences in mtDNA, although it tended to be higher in post-cardiac-arrest patients, especially those with poor neurological outcomes. Larger studies are needed, particularly in the context of severe hypoxic brain injury where additional early markers would be most useful.

## Figures and Tables

**Table 1:** Post-cardiac-arrest patients compared to healthy controls.

	Post-Cardiac-Arrest Patients (N = 86)	Healthy Controls (N = 10)	*p*-Value
Male sex ^†^	76 (88.5%)	8 (80.0%)	0.608
Age (years) ^‡‡^	66 [57–73] ^1^	68 [56–71] ^1^	0.852
BMI ^‡‡^	28 [25–31]	27 [22–29]	0.218
Charlson Comorbidity Index ^‡‡^	3 [1.8–4]	2 [1.5–3.3]	0.502
Cytochrome c (ng/mL) ^‡‡^	2.029 [0.85–4.97]	0 [0.0–0.16]	<0.001 *
mtDNA (copies/μL) ^‡‡^	95,228 [52,566–194,060]	41,466 [28,199–104,708]	0.074

Continuous data are represented as median [IQR]; categorical data are represented as number of subjects (%). mtDNA = mitochondrial DNA. * Statistically significant difference at *p* < 0.05. ^†^ *p*-values calculated using Fisher’s exact test. ^‡‡^ *p*-values calculated using Mann–Whitney U test. ^1^ All study participants were aged >18 years.

**Table 2:** Characteristics of post-cardiac-arrest patients with CPC 1–2 and CPC 3–5.

	Patients with CPC 1–2 (N = 54)	Patients with CPC 3–5 (N = 32)	*p*-Value
Male sex ^†^	47 (87.0%)	29 (90.6%)	0.738
Age (years) ^‡^	64 (13)	66 (12)	0.366
BMI ^‡‡^	27 [25–30]	29 [26–31]	0.166
Charlson Comorbidity Index ^‡‡^	2 [1–4]	3 [2–4]	0.053
OHCA ^†^	48 (88.9%)	26 (81.3%)	0.350
Primary cardiac arrest ^†^	50 (92.6%)	27 (84.4%)	0.283
Shockable initial rhythm ^††^	46 (85.2%)	18 (56.3%)	0.003 *
Cardiac arrest etiology ^†^			0.407
Acute myocardial infarction	38 (70.4%)	22 (68.8%)	
Other cardiac etiology	13 (24.1%)	5 (15.6%)	
Respiratory insufficiency	1 (1.9%)	2 (6.3%)	
Sepsis	0	1 (3.1%)	
Other	2 (3.7%)	2 (6.3%)	
Witnessed cardiac arrest ^†^	51 (94.4%)	28 (87.5%)	0.416
EMS witnessed cardiac arrest ^††^	11 (20.4%)	7 (21.9%)	0.868
BLS ^†^	46 (85.2%)	28 (87.5%)	1.000
Time to EMS (min) ^‡‡^	6 [3–9]	8 [3–12]	0.251
Cardiac arrest duration (min) ^‡‡^	17.5 [13–25.5]	30.0 [23–35]	<0.001 *
ECPR ^†^	4 (7.4%)	1 (3.1%)	0.647
PCI before admission ^††^	32 (59.3%)	21 (65.6%)	0.557
SAPS II ^‡^	54 (10.1)	61 (8)	0.002 *
APACHE II ^‡^	24 (5)	27 (5)	0.002 *
SOFA ^‡‡^	10 [7–11]	10 [9–12]	0.116
Lactate at admission (mmol/L) ^‡‡^	2.18 [1.49–4.16]	4.27 [2.24–7.32]	0.008 *
Troponin I (high sensitivity) (ng/L) ^‡‡^	1877 [448–7215]	3731 [432–20,378]	0.571
Norepinephrine at admission (mcg/kg/min) ^‡‡^	0.13 [0–0.22]	0.2 [0.09–0.37]	0.071
Dobutamine at admission (mcg/kg/min) ^‡‡^	0.00 [0–0]	0.00 [0–0]	0.273
Temperature at admission (°C) ^‡‡^	35.2 [34.6–35.8]	35 [34.5–36]	0.931
ATC with hypothermia (33 ± 0.5 °C) ^††^	18 (33.3%)	11 (34.4%)	0.921

Continuous data are represented as mean (SD) and median [IQR], categorical data are represented as number of subjects (%). APACHE II = Acute Physiology and Chronic Health Evaluation II Score; ATC = active temperature control; BLS = basic life support; BMI = body mass index; CPC = Cerebral Performance Category Score; ECPR = extracorporeal cardiopulmonary resuscitation; EMS = emergency medical services; OHCA = out-of-hospital cardiac arrest; PCI = percutaneous coronary intervention; SAPS II = Simplified Acute Physiology Score II; SOFA = Sequential Organ Failure Assessment Score. * Statistically significant difference at *p* < 0.05. ^†^ *p*-values calculated using Fisher’s exact test. ^††^ *p*-values calculated using chi-square test. ^‡^ *p*-values calculated using the independent samples Student’s *t*-test. ^‡‡^ *p*-values calculated using Mann–Whitney U test.

**Table 3:** Cytochrome c, mtDNA, and neuroprognostication results for patients with CPC 1–2 and CPC 3–5.

	Patients with CPC 1–2 (N = 54)	Patients with CPC 3–5 (N = 32)	*p*-Value
Cytochrome c (ng/mL) ^‡‡^	1.735 [0.717–3.40]	4.109 [1.149–8.457]	0.011 *
mtDNA (copies/μL) ^‡‡^	87,855 [47,598–172,464]	126,452 [69,447–260,334]	0.208
Time from ROSC to cytochrome c and mtDNA sample (min) ^‡‡^	165 [129–205]	154 [119–200]	0.583
NSE after 72 h (mcg/L) ^‡‡^	19.4 [14.5–24.5]	185.1 [27.5–256.8] ^1^	<0.001 *
Head CT ^†^			<0.001 *
Normal	17 (77.3%)	7 (24%)	
Mild HIBI	5 (22.7%)	1 (3.8%)	
Moderate to severe HIBI	0	18 (69.2%)	
EEG not very malignant ^†^	20 (100.0%) ^2^	16 (59.3%) ^3^	0.001 *
Cortical SSEP normal ^†^	12 (100.0%) ^4^	11 (55.0%) ^5^	0.012 *

Continuous data are represented as median [IQR]; categorical data are represented as number of subjects (%). CPC = Cerebral Performance Category Score; CT = computed tomography; EEG = electroencephalogram; HIBI = hypoxic–ischemic brain injury; mtDNA = mitochondrial DNA; ROSC = return of spontaneous circulation; SSEP = somatosensory evoked potentials. * Statistically significant difference at *p* < 0.05. ^†^ *p*-values calculated using Fisher’s exact test. ^‡‡^ *p*-values calculated using Mann–Whitney U test. ^1^ Five patients had NSE above the upper measurable limit of 370 mcg/L. ^2^ N = 20, ^3^ N = 27, ^4^ N = 12, ^5^ N = 20.

## Data Availability

Data are available on request.
